# Preparation of 2-Arachidonoylglycerol by Enzymatic Alcoholysis: Effects of Solvent and Water Activity on Acyl Migration

**DOI:** 10.3390/foods11203213

**Published:** 2022-10-14

**Authors:** Xiaohan Wang, Keying Liu, Yifan Wang, Zhuoneng Huang, Xiaosan Wang

**Affiliations:** 1 School of Food Science and Technology, Jiangnan University, Wuxi 214122, China; 2 National Engineering Research Center for Functional Food, Jiangnan University, Wuxi 214122, China; 3 Collaborative Innovation Center of Food Safety and Quality Control, Jiangnan University, Wuxi 214122, China

**Keywords:** 2-monoacylglycerol, enzymatic alcoholysis, acyl migration, solvent, water activity

## Abstract

Enzymatic alcoholysis was performed in an organic medium to synthesize 2-monoacylglycerol (2-MAG) rich in arachidonic acid. The results showed that solvent type and water activity (*a*_w_) significantly affected the 2-MAG yield. Under the optimum conditions, 33.58% 2-MAG was produced in the crude product in *t*-butanol system. Highly pure 2-MAG was obtained after two-stage extraction using 85% ethanol aqueous solution and hexane at first stage and dichloromethane and water at second stage. Isolated 2-MAG was used as substrate to investigate the effect of solvent type and *a*_w_ on 2-MAG acyl migration in a lipase-inactivated system. The results indicated that non-polar solvents accelerated the acyl migration of 2-MAG, whereas isomerization was inhibited in polar solvent systems. The *a*_w_ exhibited the strongest inhibition effect on 2-MAG isomerization at 0.97, but also affected the hydrolysis of glycerides and lipase selectivity.

## 1. Introduction

Structured lipids have modified structures from changing fatty acid composition and/or positional distribution by interesterification, transesterification, acidolysis, or alcoholysis [1,2,3,4]. Structural lipids have wider application and greater added-value than natural lipids. Their enhanced functionality and nutritional value, such as those rich in polyunsaturated fatty acids, can prevent neurodegenerative diseases [5,6,7]. The position of fatty acid on the glycerol backbone is an important factor affecting the physiological function and nutritional value of a structured lipid. 2-Monoacylglycerol (2-MAG) rich in polyunsaturated fatty acids has been demonstrated to have many biological functions [8]. Many studies have shown that the endogenous lipid signaling molecule 2-arachidonoylglycerol (2-AG) plays a vital role in the immune system, cardiovascular system, and central nervous system, with physiological functions including modulation of anxiety, regulation of appetite, and anti-inflammation and anti-tumor effects [9,10,11,12,13]. Thus, synthesis of 2-MAG rich in arachidonic acid (ARA) is of great practical significance.

2-MAG can be prepared by enzymatic or chemical synthesis. Chemical methods for 2-MAG synthesis require higher temperatures or longer reaction times, resulting in promotion of 2-MAG acyl migration and induction of ARA oxidation [14]. In addition, chemical synthesis has the disadvantages of low yield, extremely high raw material costs, and usage of toxic catalyst [15,16]. Thus, 2-MAG is usually prepared by lipase-mediated synthesis [4,17]. Compared to chemical synthesis, enzymatic synthesis has the advantages of milder reaction conditions, easily available raw materials, higher selectivity, limited byproducts, and less waste production, and is more suitable for production of 2-AG [18]. Enzymatic alcoholysis is an efficient way to obtain 2-AG using lipase B from *Candida antarctica* [17,19,20]. Regardless of the methods used, acyl migration, a spontaneous thermodynamic process, is inevitable in 2-MAG synthesis [20,21,22]. Generally, the fatty acid originally at the sn-2 position is spontaneously transferred to the sn-1 position to form undesirable 1-monoacylglycerol (1-MAG), resulting in decreased yield of 2-MAG and activity loss. Previous studies have shown that acyl migration reaches equilibrium at a 9:1 ratio of 1-MAG to 2-MAG [22]. Thus, to prepare 2-MAG with a high yield, it is necessary to understand the process and inhibition of acyl migration in enzymatic alcoholysis [20,23]. Many factors affect acyl migration of 2-MAG, including *a*_w_, temperature, time, type of solvent, and carrier material of the immobilized enzyme [24,25,26]. It is known that temperature and lipase carrier material accelerate acyl migration; the mechanism is relatively clear [22,25]. Few studies have reported the effects of water activity (*a*_w_) and solvent type on isomerization of partial acylglycerols [24,25,26,27]. 

In this study, the effects of the reaction medium and *a*_w_ on acyl migration in alcoholysis were investigated and the influencing mechanism was discussed. The conditions for preparation of 2-AG by lipase-catalyzed alcoholysis of *Mortierella alpina* oil were optimized, and the effects of the solvent and *a*_w_ on alcoholysis were investigated. To further study the effect of medium and *a*_w_ separately, 2-AG was purified from the crude product prepared by enzymatic alcoholysis and was used as the raw material to investigate the effects of the solvent and *a*_w_ on acyl migration in the enzyme inactivation system.

## 2. Materials and Methods

### 2.1. Materials

Refined ARA-enriched fungal oil from *M. alpina* was provided by CABIO Biotechnology Co., Ltd. (Wuhan, China) and stored at −20 °C until use. The major fatty acids in the ARA oil were ARA (46.5%), tetracosanoic acid (11.7%), palmitic acid (9.8%), stearic acid (6.3%) and oleic acid (6.1%). Lipozyme 435 (immobilized lipase B from *Candida antarctica*, immobilized on acrylic resin, 10 U/mg), Lipozyme RM IM (1,3-specific lipase from *Rhizomucor miehei*, immobilized on ion-exchange resins, 275 IUN/g), and Lipozyme TL IM (immobilized 1,3-specific lipase from *Thermomyces lanuginosus*, immobilized on silica gel, 30 U/g) were generously donated by Novozymes (Beijing, China). Lipase CL “Amano” IM (immobilized lipase B from *Candida antarctica*, 400 U/g), Lipase DF “Amano” IM (immobilized 1,3-specific lipase from *Rhizopus oryzae*, 600 U/g), Lipase DF “Amano” 15 (free 1,3-specific lipase from *Rhizopus oryzae*, 150 U/mg), Lipase AY “Amano” S (free lipase from *Candida cylindracea*, 30 U/mg), and Lipase G “Amano” 50 (free lipase from *Penicillium camemberti*, 50 U/mg) were generously donated by Amano Enzyme, Inc. (Wuxi, China). One lipase unit (U) was defined as the amount of lipase producing 1 μmol of fatty acid per min at 30 °C at pH 7.0 using triolein as substrate. Specific activity was defined as units per milligram of protein. 1-monopalmitin and 2-monopalmitin standards were obtained from Sigma-Aldrich Chemical Co., Ltd. (Shanghai, China). HPLC-grade hexane, isopropyl alcohol, and methanoic acid were obtained from Beijing J & K Scientific Company (Beijing, China). Other analytical-grade organic solvents were purchased from Sinopharm Chemical Reagent Company (Shanghai, China).

### 2.2. Preparation of 2-MAG by Enzymatic Alcoholysis at a Controlled a_w_

Reactants including 2 g of fungal oil and 4 g of ethanol (molar ratio of 1:40, oil to ethanol) were mixed in a 25-mL glass vessel with magnetic agitation at 400 rpm. Lipase was used as a biocatalyst to start the reaction. Alcoholysis was performed at a controlled *a*_w_; additional solvent was added to the reaction system to investigate the effect of solvent type. After completion of alcoholysis, the product was centrifuged at 4000 rpm for 4 min to fully remove the lipase. The solvents were removed by rotary evaporation at 40 °C to obtain the final alcoholysis product for HPLC analysis. 

To maximize 2-MAG content in the crude product, eight commercial lipases used in the alcoholysis system were evaluated and screened in the following conditions: 1:40 molar ratio of oil to ethanol, lipase load of 8% or 15% (relative to mass of oil), *a*_w_ of 0.53, 35 °C, without additional solvent. The effects of solvent type, solvent quantity, *a*_w_, alcoholysis temperature, and time on 2-MAG content in the crude product were studied. The solvents included ethanol, acetone, *t*-butanol, dichloromethane, and hexane. The solvent quantity ranged from 1 mL to 4 mL; *a*_w_ ranged from 0.11 to 0.97. The alcoholysis temperature and time ranged from 25 °C to 40 °C and from 2 h to 8 h, respectively. After a factor optimization was completed, the optimized value was used for the next factor optimization.

During enzymatic alcoholysis, the *a*_w_ of the reaction system was accurately controlled using a previous method [28]. A certain *a*_w_ was achieved by equilibrating the reaction mixture and lipase with an aqueous saturated salt solution at room temperature for 24 h in two separated desiccators. The saturated salt solution had a known *a*_w_: 0.11 for saturated LiCl solution, 0.53 for saturated Mg(NO_3_)_2_ solution, 0.74 for saturated NaNO_3_ solution, and 0.97 for saturated K_2_SO_4_ solution. 

### 2.3. Purification of Crude Product to Prepare High Purity 2-MAG

After removal of the lipase and solvent, fatty acid ethyl ester (FAEE), diacylglycerol (DAG), and triacylglycerol (TAG) in the crude product were removed by solvent extraction to obtain an 85% ethanol aqueous solution containing 2-MAG according to the method used by Zhang et al. [4]. In addition to 2-MAG, the polar ethanol solution may also contain trace amounts of lipase protein and glycerol. Thus, further purification was required. Purified 2-MAG was obtained after removal of water and ethanol from the polar phase. Purified 2-MAG (3 g) was mixed with 100 mL of dichloromethane. An equal amount of distilled water was added for extraction. After two layers were observed, a dichloromethane layer containing 2-MAG was collected. The solvent was removed by evaporation under reduced pressure at 25 °C to obtain the final purified 2-MAG product, which was stored in a refrigerator at −20 °C for further use. The isolated yield of 2-MAG was calculated according to the ratio of the mass of isolated 2-MAG to the theoretical mass of 2-MAG.

### 2.4. Acyl Migration of 2-MAG in Lipase-Inactivated System

To eliminate interference of other factors and investigate the effects of solvent type and *a*_w_ on the acyl migration of 2-MAG, a lipase-inactivated system was used. Purified 2-MAG (0.7 g) and inactivated Lipozyme TL IM (0.3 g) were mixed with 7 mL of solvent (ethanol, acetone, *t*-butanol, dichloromethane, or hexane). Acyl migration was conducted at 30 °C in a sealed vessel containing a saturated salt solution of LiCl, Mg(NO_3_)_2_, NaNO_3_, or K_2_SO_4_ with an *a*_w_ of 0.11, 0.53, 0.74, or 0.97, respectively, for the reaction system. Periodically, 100-μL samples were collected from the reaction crude products and analyzed by a high-performance liquid chromatography refractive index detector (HPLC–RID) after adding 1 mL of mobile phase solvent. 

To ensure that the lipase was totally inactivated, alcoholysis was conducted in the following conditions: 1:40 molar ratio of oil to ethanol, 15% inactivated Lipozyme TL IM, 3 mL *t*-butanol as solvent, *a*_w_ of 0.53, and 30 °C. After 24 h of reaction, samples were collected from the crude product and analyzed by HPLC–RID. We concluded that the lipase was totally inactivated when no FAEE or partial acylglycerol was observed.

### 2.5. Analysis of Alcoholysis Product by HPLC–RID

Identification and quantification of the reaction crude product and purified 2-MAG were determined by HPLC–RID (Waters Corp., Milford, MA, USA) using a Sepax HP-Silica column (particle size of 5 μm, 4.6 mm × 250 mm, Sigma-Aldrich Corp., K.K., Tokyo, Japan). The samples were eluted with solvent at 1.0 mL/min and the column temperature was maintained at 30 °C. The solvent was a mixture containing hexane, isopropanol, and methanoic acid with a ratio of 15:1:0.003 (*v*/*v*/*v*). The glyceride standards were used to identify the HPLC peaks of the samples. The standard curve obtained from the peak areas and the standard concentrations were used to quantify the glyceride product. All reactions and their corresponding analyses were carried out in triplicate.

### 2.6. Statistical Analysis

All data were analyzed by one-way analysis of variance (one-way ANOVA) using Origin 2018 (Origin Lab, Northampton, MA, USA). Tukey’s test was conducted to determine significant differences at the *p* < 0.05 level. Data presented in the figures represent average values with standard deviations.

## 3. Results and Discussion

### 3.1. Effect of Lipase Type and Load on Alcoholysis

**Figure 1:**
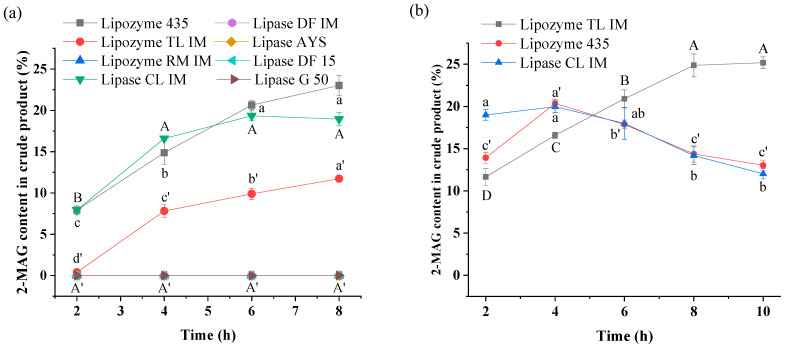
Effects of lipase type on the content of 2-MAG in the crude product with 8% (**a**) and 15% lipase load (**b**). Reaction conditions: (**a**) molar ratio of 1:40 (fungal oil/ethanol), lipase load of 8% (relative to the mass of oil), *a*_w_ of 0.53, 35 °C; (**b**) molar ratio of 1:40 (fungal oil /ethanol), lipase load of 15% (relative to the mass of oil), *a*_w_ of 0.53, 35 °C. Different letters in the figure indicated significant differences at the *p* < 0.05 level.

Eight lipases from two enzyme preparation companies exhibited different catalytic activities in alcoholysis for synthesis of 2-MAG. When the added amount of lipase (relative to the mass of oil) was 8%, as shown in Figure 1a, three immobilized lipases, Lipozyme 435, Lipozyme TL IM, and Lipase CL IM exhibited considerably higher catalytic activities than the other lipases. Lipozyme 435 and Lipase CL IM were derived from *Candida antarctica* lipase B; the 2-MAG contents in the crude product reached 23.02% and 19.34%, respectively, after 8 h with these lipases as biocatalysts. In the Lipozyme TL IM-catalyzed reaction system, 11.73% 2-MAG was produced in the crude product. For other lipases as catalysts, no 2-MAG was produced in the system with ethanol as substrate. In this study, 1 mol TAG can theoretically be converted to 2 mol FAEE and 1 mol 2-MAG. Thus, the maximum 33.3% molar content of 2-MAG in the crude product is equal to a 100% 2-MAG yield.

Previous studies have demonstrated that lipases from Lipozyme 435 and Lipozyme TL IM show strong 1,3-specificity in alcoholysis [29,30]. In this study, we also found that another commercial lipase from *Candida antarctica* lipase B (Lipase CL IM) also had 1,3-specificity in alcoholysis. Thus, these are suitable biocatalysts to prepare 2-MAG. Lipozyme RM IM, Lipase DF IM, and Lipase DF 15 were also considered as 1,3-specific lipases in acidolysis [31] but showed no catalytic activities in alcoholysis, indicating that they may have low tolerance to polar ethanol [32,33]. This result is in agreement with results from our previous study [34]. 

According to the results in Figure 1a, Lipozyme 435 seemed to be the best choice for preparation of 2-MAG by alcoholysis, followed by Lipozyme TL IM. A low 2-MAG content in the Lipozyme TL IM-mediated system may be attributed to a low lipase load. Thus, these three lipases were selected for further experiments with the lipase load increased to 15%. Figure 1b shows that the highest 2-MAG content (24.87%) obtained in the crude product after 8 h was observed in the Lipozyme TL IM-catalyzed system, considerably higher than the contents in Lipozyme 435- and Lipase CL IM-mediated systems. The 2-MAG content in alcoholysis product obtained with Lipozyme 435 or Lipase CL IM as biocatalysts reached the maximum at 4 h before decreasing, possibly due to a high load of immobilized material and a long reaction time, increasing the probability of 2-MAG isomerization to 1-MAG, which was further converted to FAEE and glycerol by a 1,3-specific lipase, ultimately leading to a decrease in 2-MAG yield [20]. Compared to Lipozyme 435 and Lipase CL IM (approximately 2300 USD/kg), Lipozyme TL IM is inexpensive (approximately 100 USD/kg). In previous 2-MAG synthesis reactions, most studies used catalytic efficiency as a measure and lipase B from *Candida antarctica* as the optimum lipase to catalyze the alcoholysis reaction [17,19,20,29]. However, in this study, the same catalytic efficiency was obtained with 8% Lipozyme 435 and 15% Lipozyme TL IM. With this in mind, the cost of the reaction was considered; 15% Lipozyme TL IM was used for subsequent optimization. 

### 3.2. Optimization of Reaction Conditions for Enzymatic Alcoholysis

#### 3.2.1. Effect of Solvent Type on Enzymatic Alcoholysis

**Figure 2:**
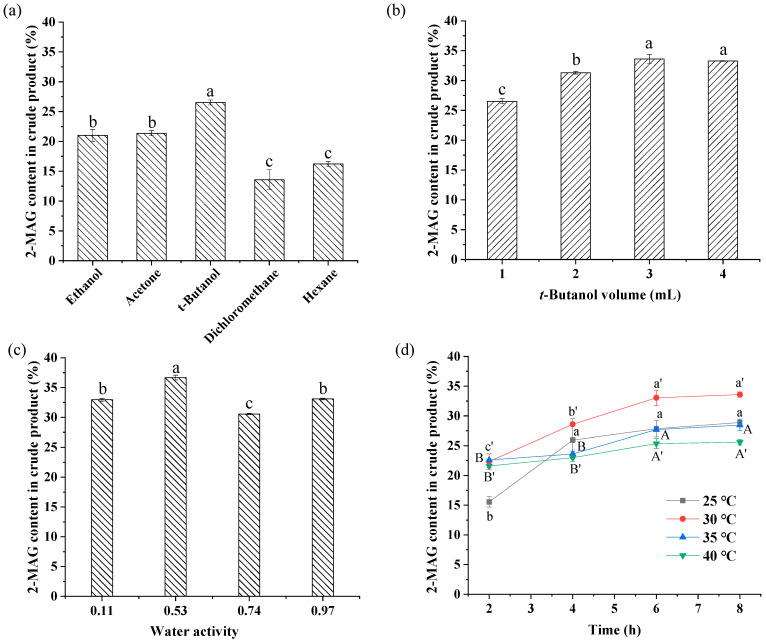
Optimization of rea ction conditions of enzymatic alcoholysis. Reaction conditions: (**a**) molar ratio of 1:40 (fungal oil/ethanol), 15% (relative to the mass of oil) Lipozyme TL IM, solvent volume of 1 mL, *a*_w_ of 0.53, 35 °C, 8 h. (**b**) molar ratio of 1:40 (fungal oil/ethanol), 15% (relative to the mass of oil) Lipozyme TL IM, *t*-butanol as solvent, *a*_w_ of 0.53, 35 °C, 8 h. (**c**) molar ratio of 1:40 (fungal oil/ethanol), 15% (relative to the mass of oil) Lipozyme TL IM, 3 mL *t*-butanol as solvent, 35 °C, 8 h. (**d**) molar ratio of 1:40 (fungal oil/ethanol), 15% (relative to the mass of oil) Lipozyme TL IM, 3 mL *t*-butanol as solvent, *a*_w_ of 0.53. Different letters in the figure indicated significant differences at the *p* < 0.05 level.

Five solvents with log P ranging from −0.24 to 3.5 were selected to investigate the effect of different solvents on enzymatic alcoholysis. The log P values for ethanol, acetone, *t*-butanol, dichloromethane, and hexane are in descending order. In the reaction of enzymatic preparation of 2-MAG, organic solvent, as a reaction medium, usually affects the catalytic activity [35] and the acyl migration of partial glycerides in the reaction, which affects the 2-MAG yield. Thus, the effect of solvent type was optimized in this study. The optimized reaction conditions are presented in Figure 2a.

Figure 2a shows that solvent type had a significant effect on the content of 2-MAG. In the *t*-butanol system with moderate polarity, the 2-MAG content reached 26.53%, considerably higher than contents obtained in other solvent systems. The lowest 2-MAG contents in the crude product were observed in non-polar media (dichloromethane and hexane). This result is consistent with results reported by Damstrup et al. [36] in synthesizing MAG by glycerolysis with Lipozyme TL IM as biocatalyst in 13 solvent systems. In that study, *t*-butanol effectively improved the poor miscibility of reactants between glycerol and oil, leading to an increased MAG yield in the *t*-butanol medium. However, in this study, improvement of reactant miscibility was not the only cause of increased 2-MAG content in the *t*-butanol system; hexane and dichloromethane also improved reactant miscibility between ethanol and oil. The effects of solvent polarity on acyl migration of 2-MAG and lipase activity also play a crucial role in enzymatic alcoholysis [17,37]. Based on the results, *t*-butanol was chosen as the best solvent for further optimization.

#### 3.2.2. Effect of Added Amount of *t*-Butanol on Enzymatic Alcoholysis 

In the alcoholysis system, ethanol acts as a reaction substrate and the medium. Addition of *t*-butanol was conducive to improving the 2-MAG yield from alcoholysis. Thus, it was necessary to determine the optimal amount of added *t*-butanol. It is observed in Figure 2b that the 2-MAG content increased with an increase in *t*-butanol volume ranging from 1 mL to 3 mL. Overall, the addition of *t*-butanol increased the yield. A large amount of added *t*-butanol may be beneficial for inhibition of acyl migration of 2-MAG or for enhancing the solubility of reactant and product [17]. However, no significant differences in 2-MAG content were found with 3 mL and 4 mL of solvent. For environmental protection, use of solvents should be minimized. Thus, 3 mL of *t*-butanol was considered as the optimum added amount.

#### 3.2.3. Effect of *a*_w_ on Enzymatic Alcoholysis

Generally, *a*_w_ has a large impact on acyl migration of partial glycerides and enzyme activity [22]. However, few studies have reported the effect of *a*_w_ on the enzymatic reaction. Several studies mentioned the effect of *a*_w_ on the acidolysis and esterification reactions [38,39]. The effect on the alcoholysis reaction is still uncertain. This study is the first to consider the effect of *a*_w_ on the enzymatic alcoholysis reaction. Figure 2c compares the 2-MAG content in crude alcoholysis products with *a*_w_ ranging from 0.11 to 0.97. It is observed that the 2-MAG content is related to the *a*_w_ of the system. When *a*_w_ was increased from 0.11 to 0.53, the 2-MAG content increased from 32.98% to 36.68%. As *a*_w_ was increased to 0.74, the 2-MAG content decreased to 30.57%. Increasing the *a*_w_ to 0.97 increased the 2-MAG content to 33.11%, lower than that at 0.53. Thus, 0.53 was selected as the most appropriate *a*_w_ for alcoholysis. 

#### 3.2.4. Effect of Temperature and Time on Enzymatic Alcoholysis

Figure 2d shows the change in 2-MAG content of the crude product at different temperatures over time. Increasing the reaction temperature generally increases the mass transfer, improves the reaction rate, and shortens the reaction time. However, excessive temperature may reduce the activity and stability of the lipase. In Figure 2d, when the reaction temperature was increased from 25 °C to 30 °C, the 2-MAG content increased from 28.89% to 33.58%. Increasing temperature further from 30 °C to 40 °C resulted in a continuous decrease in 2-MAG content, possibly attributed to an increased rate of acyl migration caused by a relatively high temperature [21,22]. Moreover, ARA-rich fungal oil with many unsaturated double bonds is vulnerable to temperature. MAG synthesis must be conducted at a relatively low temperature; 30 °C was the ideal temperature for 2-MAG synthesis. 

At 30 °C, with prolonged reaction time, the sn-1(3) fatty acids of TAG were cleaved by lipase, leading to formation of 2-MAG. After reaction for 6 h, the alcoholysis reached equilibrium. There was no significant difference in 2-MAG content at 6 h and 8 h. According to Figure 2d, 6 h was considered as the optimum reaction time. In these conditions, 33.05% 2-MAG was formed in the crude product. After purification by solvent extraction, impurities including FAEE, DAG, and TAG were removed and the 2-MAG purity reached 98.5%. The purified 2-MAG was used as substrate for the subsequent acyl migration study. 

### 3.3. Effect of Solvent Type and a_w_ on 2-MAG Isomerization in Catalyst-Free System

**Figure 3:**
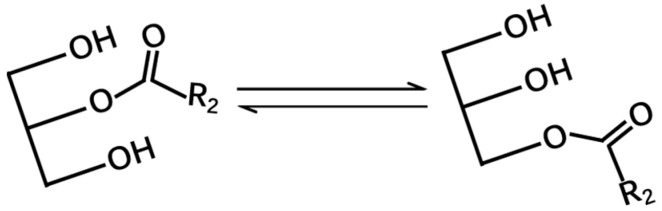
The migration of 2-MAG to 1-MAG.

From the results, solvent type and *a*_w_ had strong effects on Lipozyme TL IM-catalyzed alcoholysis, possibly attributed to their effects on acyl migration of 2-MAG and lipase activity during the reaction [24,27]. With the involvement of acyl migration, the enzymatic alcoholysis has multiple reactions occurring simultaneously, and it is difficult to directly evaluate the intensity of several acyl migration reactions. To confirm our speculation and to study how solvent type and *a*_w_ affect 2-MAG isomerization, the acyl migration of 2-MAG was chosen as a representative and a catalyst-free system was established by totally inactivating the lipase. The catalyst-free acyl migration system consisted of inactivated Lipozyme TL IM, solvent, and purified MAG with concentrations similar to those of the crude product. The acyl migration was conducted at 30 °C, consistent with the optimum conditions of the alcoholysis reaction, to simulate as much as possible the environment of the enzymatic alcoholysis. The acyl migration of 2-MAG in this system is described in Figure 3. The change in the ratio of 2-MAG to total MAG with time was investigated in the lipase-inactivated system, as shown in Figure 3. A lower ratio of 2-MAG to total MAG indicates more 2-MAG migrating to 1-MAG and a faster acyl migration rate. 

#### 3.3.1. Effect of Solvent Type on 2-MAG Isomerization

**Figure 4:**
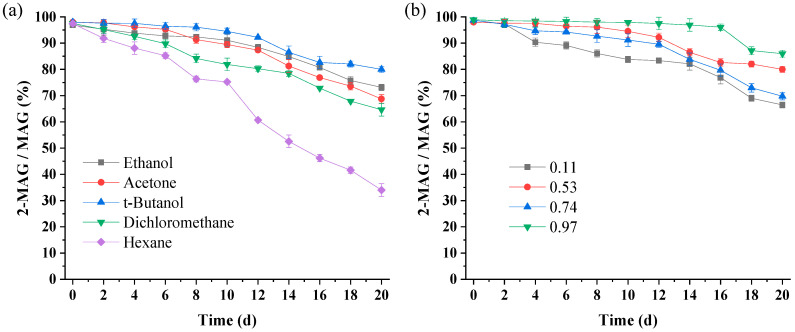
Acyl migration of 2-MAG in various solvents (**a**) and *a*_w_ (**b**) at 30 °C for 20 days. Acyl migration conditions: (**a**) 0.7 g of purified 2-MAG, 7 mL of solvent, 0.3 g inactivated Lipozyme TL IM, *a*_w_ of 0.53; (**b**) 0.7 g of purified 2-MAG, solvent of 7 mL *t*-butanol, 0.3 g inactivated Lipozyme TL IM.

With the lipase totally inactivated and *a*_w_ maintained at 0.53, only the solvent affects 2-MAG isomerization. According to Figure 4a, after 20 days, the 2-MAG/MAG ratios in the ethanol, acetone, *t*-butanol, dichloromethane, and hexane systems were 73.14%, 68.76%, 79.98%, 64.62%, and 34.01%, respectively, indicating that *t*-butanol exhibited the best inhibitory effect on acyl migration with time, followed by ethanol and acetone. Non-polar dichloromethane and hexane, especially hexane with the lowest polarity, strongly promoted acyl migration. These results suggest that acyl migration of 2-MAG was closely related to solvent polarity (log P). However, the log P of the solvent is not the only parameter determining the acyl migration rate; acyl migration of 2-MAG in ethanol (log P of −0.24) and acetone (log P of −0.23) were faster than in *t*-butanol (log P of 0.6) (Figure 4a). Generally, the more polar the solvent, the stronger the inhibition of acyl migration. 

Acyl migration is a nucleophilic substitution reaction [40]. Negatively charged hydroxyl oxygen at the sn-1 position of 2-MAG has lone pair electrons, which nucleophilic attack on the positively charged carbon, leading to the formation of a five-membered ring intermediate [40,41,42]. Subsequently, the hydroxyl oxygen again makes a nucleophilic attack on the carbon, the strained ring opens and 1-MAG is formed [24]. The high-polarity systems are not conducive to charge dispersion in the transition state and the energy of the transition state increased, leading to a reduction in the acyl migration rate [20,24]. While slower acyl migration occurred in *t*-butanol with higher log P value than ethanol and acetone (Figure 4a). Damstrup et al. [36] have pointed out that both polarity and functional groups play an important role in the performance of solvents. Thus, the inhibition of *t*-butanol may be due to its special functional groups, which increase the activation energy of acyl migration [24].

**Figure 5:**
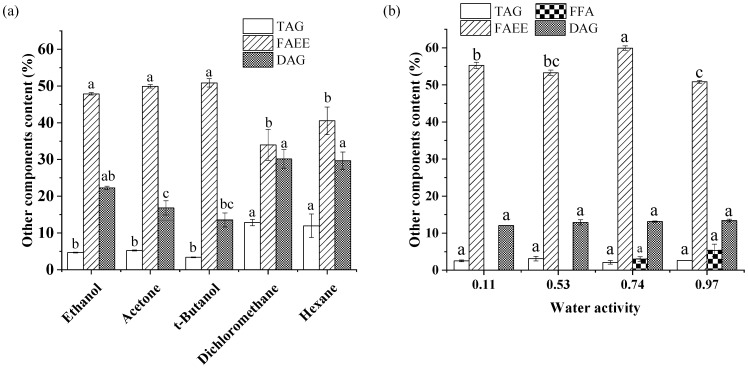
Effect of solvent type (**a**) and *a*_w_ (**b**) on the contents of other lipid components in the crude product. Reaction conditions: (**a**) molar ratio of 1:40 (fungal oil/ethanol), 15% (relative to the mass of oil) Lipozyme TL IM, solvent volume of 1 mL, *a*_w_ of 0.53, 35 °C, 8 h; (**b**) molar ratio of 1:40 (fungal oil/ethanol), 15% (relative to the mass of oil) Lipozyme TL IM, 3 mL *t*-butanol as solvent, 35 °C, 8 h. Different letters in the figure indicated significant differences at the *p* < 0.05 level.

The effect of solvent type on the other lipid components of the crude product is shown in Figure 5a. The main impurity in the alcoholysis product was FAEE, followed by DAG and TAG. TAG contents of crude product in the dichloromethane and hexane systems were 12.84% and 11.96%, respectively, several times greater than those in the ethanol, acetone, and *t*-butanol systems. Correspondingly, FAEE levels in nonpolar systems were lower, indicating that the alcoholysis of glycerides progressed slowly in these two systems. Theoretically, the miscibility of oil with a low-polar solvent is better than with a polar solvent; the contact areas between enzyme and substrate and between substrate and substrate are larger [43], which usually leads to improved catalytic efficiency of the enzyme. Surprisingly, increasing the miscibility of the oil with ethanol in a non-polar system led to a decreased 2-MAG yield in lipase-catalyzed alcoholysis. There are two explanations for this observation. First, the low-polar solvents strongly promoted acyl migration of 2-MAG to 1-MAG, which was subsequently converted to glycerol and FAEE by Lipozyme TL IM. In addition, in organic solvents, the substrate must be separated from the solvent before binding to the active center of the lipase (desolvation) [44]. The hydrophobic matrix oil binds more closely to more nonpolar solvents, making it difficult to “squeeze out” of the solvents, hindering interaction between the substrate and the lipase, reducing the catalytic efficiency of the lipase. Thus, only approximately 15% 2-MAG was produced in dichloromethane and hexane systems in Lipozyme TL IM-catalyzed alcoholysis after 8 h (Figure 2a).

#### 3.3.2. Effect of *a*_w_ on 2-MAG Isomerization

The effect of *a*_w_ on the 2-MAG yield in Lipozyme-catalyzed alcoholysis may also be attributed to its effect on acyl migration. When the lipase was totally inactivated and the solvent was *t*-butanol, only *a*_w_ affected 2-MAG isomerization. According to the change trend of the 2-MAG/MAG ratio over 20 days shown in Figure 4b, the rate of acyl migration for different *a*_w_ values can be ranked corresponding to 0.11 > 0.74 > 0.53 > 0.97. The highest acyl migration rate occurred in the 0.11 *a*_w_ system. Increasing *a*_w_ led to a decrease in acyl migration in the catalyst-free system. Many studies have shown that the polarity of the system increases with an increase in *a*_w_, which is not conducive to charge dispersion of the acyl migration transition state, inhibiting acyl migration [17,27,39] With *a*_w_ ranging from 0.53 to 0.97, a free fatty acid hydrolysis product was formed in the crude product, indicating that hydrolysis occurred at a high *a*_w_ level. 

In the enzymatic preparation of 2-MAG, the lipase must maintain a certain degree of flexibility to reach the required conformation in the state of the greatest fit with the substrate [28]. Duan et al. [28] indicated that the presence of water could make the stable closed structure of the enzyme “loose”, enhancing the flexibility of the enzyme molecule and improving the catalytic activity. However, as shown in Figure 5b, the FAEE content in the product with an *a*_w_ of 0.11 was considerably higher than that in the system with an *a*_w_ of 0.53, indicating that the main reason for the change in 2-MAG content in different *a*_w_ conditions may not be the change in enzyme activity. With a continuous increase in *a*_w_, excessive water pushed the enzyme molecules past the optimal state; the structure became too soft and loose, weakening the catalytic activity of the enzyme [44]. When *a*_w_ was increased to 0.97, inhibition of acyl migration of 2-MAG was beneficial to the increase in 2-MAG yield in enzymatic alcoholysis. However, the lowest FAEE level indicated that an excessively high *a*_w_ decreased enzyme activity, producing a less favorable environment than with an *a*_w_ of 0.53. In the 0.53 *a*_w_ system, the enzyme was in the best hydration state and the acyl migration was relatively slow; the highest 2-MAG content was observed in Lipozyme TL IM-catalyzed alcoholysis.

#### 3.3.3. Acyl Migration of 2-MAG in Lipase-Catalyzed and Lipase-Inactivated Systems

In the lipase-catalyzed system, when 2-MAG is migrated to 1-MAG, the produced 1-MAG is continuously converted to FAEE and glycerol by the 1,3-specific lipase (Lipozyme TL IM), resulting in a decreased level of 1-MAG or total MAG. Thus, acyl migration of 2-MAG in lipase-catalyzed system leads to the formation of glycerol and FAEE rather than 1-MAG. The ratio of 2-MAG to MAG cannot be an indicator to reflect the degree of acyl migration in enzymatic alcoholysis, whereas a high level of FAEE in the product may be attributed to enhanced alcoholysis of TAG by the sn-1,3 lipase rather than acyl migration. Additionally, sn-1,3 selectivity of the lipase in lipase-catalyzed system also varies with the solvent type and *a*_w_ [44,45]. When solvent type or *a*_w_ may make the sn-1,3 specific lipase has higher catalytic activity toward the fatty acid at the sn-2 position (becoming non-specific), the non-specific lipase can convert 2-MAG directly to FAEE in the presence of ethanol. Therefore, a low 2-MAG content in the product in enzymatic alcoholysis may be attributed to increased non-specificity of the lipase rather than acyl migration of 2-MAG. For these reasons, the influences of solvent type and *a*_w_ on the enzymatic alcoholysis were discussed by separately observing the effects on the acyl migration of 2-MAG in the catalyst-free system. The degree of migration can be evaluated by calculating the ratio of 2-MAG to MAG in the catalyst-free system. However, the actual acyl migration in lipase-catalyzed system is more complicated than that in the catalyst-free system. In non-enzymatic system, the effects of solvent type and *a*_w_ on acyl migration can be observed more visually and accurately, but the role of lipase was ignored. Based on the study in the lipase-inactivated system, we can conclude that different solvents and *a*_w_ significantly affect the acyl migration of 2-MAG in lipase-mediated alcoholysis, but influencing degree is still unknown. In further study, the influencing mechanism of the synergistic effect of lipase and acyl migration should be investigated by other evaluative measures such as kinetic parameters rather than product concentration.

## 4. Conclusions

2-AG was successfully prepared by efficient enzymatic alcoholysis. After optimization of the alcoholysis conditions, the maximum 2-MAG content in the crude product was 33.58%, and the 2-MAG purity reached 98.5% after purification. Solvent type and *a*_w_ had considerable effects on alcoholysis. They also affected the acyl migration of 2-MAG in the catalyst-free system. 2-MAG migrated much faster in non-polar solvents than in polar solvents. *A*_w_ had multiple effects on enzymatic alcoholysis. Overall, a high *a*_w_ is beneficial for inhibiting acyl migration of 2-MAG.

## Data Availability

The data presented in this study are available on request from the corresponding author.
